# Serum Trace Elements Profile in Graves' Disease Patients with or without Orbitopathy in Northeast China

**DOI:** 10.1155/2018/3029379

**Published:** 2018-01-10

**Authors:** Yongping Liu, Shanshan Liu, Jinyuan Mao, Sichen Piao, Jing Qin, Shiqiao Peng, Xiaochen Xie, Haixia Guan, Yushu Li, Zhongyan Shan, Weiping Teng

**Affiliations:** ^1^Department of Endocrinology and Metabolism, Institute of Endocrinology, Liaoning Provincial Key Laboratory of Endocrine Diseases, The First Affiliated Hospital of China Medical University, China Medical University, No. 155, North Nanjing Street, Heping District, Shenyang, China; ^2^Department of Emergency, No. 202 Hospital of People's Liberation Army, No. 5, Guangrong Street, Heping District, Shenyang, China

## Abstract

**Objective:**

The purpose of the present study was to investigate serum trace elements in Graves' disease (GD) patients with or without orbitopathy in Northeast China.

**Methods:**

Patients with newly diagnosed Graves' disease (HyGD) (*n* = 66), GD patients with euthyroid status or subclinical thyroidism after treatment (EUGD) (*n* = 55), GO patients with euthyroid status or subclinical thyroidism after treatment (GO) (*n* = 57), and normal controls (NC) (*n* = 66) were enrolled in this study. Serum trace elements were measured with ICP-MS.

**Results:**

Serum selenium (Se) levels in EUGD group (median: 7.53 *µ*g/dL), HyGD group (median: 6.76 *µ*g/dL), and GO group (median: 7.40 *µ*g/dL) were significantly lower than those in NC group (median: 9.20 *µ*g/dL, all *P* < 0.01). Serum copper (Cu) levels in GO group (median: 95.93 *µ*g/dL) were significantly lower than those in the NC group (median: 113.59 *µ*g/dL, *P* = 0.015). After being adjusted for multivariables, thyroid-specific antibodies grade was associated with low Se levels. Hyperthyroidism and thyroid-specific antibodies grade were associated with high Cu levels. In addition, orbitopathy was associated with low Cu levels.

**Conclusions:**

Thyroid autoimmunity was associated with low Se levels. Hyperthyroidism and thyroid autoimmunity may be associated with relatively high serum Cu levels. Alternatively, ophthalmopathy may be related to low serum Cu levels.

## 1. Introduction

The morbidity of Graves' disease (GD), one of the most common autoimmune thyroid diseases (AITD) [1], has been gradually increasing in China in recent years [2]. This disease is characterized by excessive thyroid hormone, hyperplastic glands, and an increase in stimulating autoantibodies to the TSH receptor (TRAb) [3]. Graves' ophthalmopathy (GO), which potentially changes the patient's appearance, affects vision, impairs the quality of life, and influences social and psychological functions, is the most frequent external thyroid manifestation of GD [4]. However, the etiopathogenesis of GD and GO is not yet completely understood. In addition to genetic and environmental factors, trace elements may play key roles in thyroid physiology and pathology [5].

Trace elements, such as selenium (Se) and copper (Cu), have been reported to be essential cofactors of antioxidant and anti-inflammation system [6, 7], which are involved in the production of thyroid hormone [8, 9]. Therefore, the trace elements in the body should be present in appropriate concentrations, and abnormal levels of trace metals can develop when the oxidation-antioxidation system fails [10, 11]. It has been reported that thyroid gland is the organ with the highest levels of trace elements [12]. These elements have potential links with thyroid hormone metabolism, and any deficiency or excess can affect thyroid hormones homeostasis [3, 13–15]. However, it has been reported that thyroid hormone can also affect trace element metabolism [16, 17]. In addition, the effects of microelements are markedly dependent on one another; for instance, low concentrations of selenium may aggravate myxedematous cretinism caused by a deficiency of iodine [18].

However, as far as we know, few studies have examined serum trace elements in patients with thyroid disease in China. In this study, we aim to compare the serum levels of trace elements, including Se, vanadium (V), iron (Fe), cobalt (Co), Cu, zinc (Zn), rubidium (Rb), strontium (Sr), and cesium (Cs), in GD patients with or without orbitopathy in Shenyang, a region with adequate iodine intake in Northeast China.

## 2. Materials and Methods

### 2.1. Subjects

One hundred and seventy-eight patients, including 55 GD patients without orbitopathy, who were treated with antithyroid drugs alone and achieved a stable euthyroid status or subclinical hyperthyroidism (EUGD group), 66 newly diagnosed hyperthyroid GD patients without GO (HyGD group), and 57 patients with mild-to-moderate GO with an euthyroid state or subclinical hyperthyroidism after treatment with antithyroid drugs (GO group), from the outpatient clinics of the Endocrine Department, the First Hospital of China Medical University, were enrolled in the present study. In addition, 66 healthy controls from the medical examination center (NC group) were enrolled. Across the groups, the subjects were matched for age, gender, and body mass index (BMI). The study was approved by the Ethics Institutional Review Board of China Medical University prior to subject recruitment. All of the participants provided written informed consent prior to enrollment.

Baseline characteristics of all participants, including age, gender, BMI, ethnicity, residence, medical history, previous thyroid treatment, current thyroid treatment, and drug history, were recorded at the interview. The eligibility criteria were as follows: between 18 and 65 years of age and no serious disease (acute infections, stroke, myocardial infarction within 6 months, diabetes, heart disease, renal or hepatic impairment, autoimmune disease, bleeding disorder, or cancer) or pregnancy. None of them have taken multivitamin with trace elements before the study. Additionally, for inclusion in this study, participants were required to comply with the following admission and inclusion criteria.

The admission criteria of HyGD included GD without clinical signs or symptoms of thyroid orbitopathy diagnosed by increased serum concentrations of free thyroxine (FT4) and triiodothyronine (FT3), positive tests for TSH-receptor antibodies (TRAb), increased I^131^ intake, and thyrotoxicosis associated with diffuse goiter. The patients with a state of euthyroidism or subclinical hyperthyroidism after treatment with antithyroid drugs who once suffered from GD were enrolled in the EUGD group. The inclusion criteria for GO included a diagnosis of mild-to-moderate GO upon examination by an experienced ophthalmologist according to the EUGOGO classification [19], a medical history of GD, and a status of euthyroidism or subclinical hyperthyroidism after treatment with antithyroid drugs. Healthy subjects without chronic and/or infectious diseases were recruited from the medical examination center as controls.

### 2.2. Methods

Elbow venous blood were collected between 8 AM and 9 AM after fasting for at least 8 hours. The sera were obtained after centrifugation and stored at −80°C prior to analysis.

Serum concentrations of trace elements were measured with inductively coupled plasma mass spectrometry (ICP-MS) (Series 7700, Agilent Technologies Inc., California, USA) as previously described [20]. Briefly, blood serum was diluted with nitric acid, and digestion was carried out using the following steps: 1000 W (0–120°C) for 15 min, 1000 W (120°C) for 2 min, 1000 W (120–180°C) for 15 min, and 1000 W (180°C) for 10 min. Next, the appropriate determination parameters for ICP-MS were chosen to detect trace elements. A standards curve and an Internal Standard Mix was used for detection. The coefficient of variation (CV) within the analyses was less than 10%.

Serum thyroid stimulating hormone (TSH), FT3, FT4, thyroperoxidase antibody (TPOAb), and antithyroglobulin antibody (TgAb) were measured using a Chemiluminescent Microparticle Immunoassay (Abbott Laboratories, Abbott Park, IL 60064, USA), and TRAb were measured using an electrochemiluminescence immunoassay with Cobas Eless 601 (Roche Diagnostics). Quality control analyses were performed before, during, and after testing. The reference ranges (0.35–4.94 mIU/L for TSH, 2.63–5.70 pmol/L for FT3, 9.01–19.05 pmol/L for FT4, 0.00–5.61 IU/mL for TPOAb, 0.00–4.11 IU/mL for TgAb, and 0.00–1.75 IU/L for TRAb) were provided by the manufacturer. The intra-assay CV for TSH were 1.1–5.0%, for FT3 were 1.4–3.4%, for FT4 were 2.3–5.3%, for TPOAb were 1.8–9.5%, for TgAb were 1.7–6.6%, and for TRAb were 0.9–7.6%.

### 2.3. Statistical Analysis

Statistical analysis was carried out using SPSS 22.0 software (SPSS Inc., Chicago, IL, USA) for Windows. Normally distributed data were expressed as the mean ± standard deviation (M ± SD), while data with abnormally distributions were expressed as the median and interquartile intervals (IQRs). One-way analysis of variance (ANOVA) or nonparametric Kruskal-Wallis tests were used to detect differences in the variables across the groups. Spearman correlation coefficients were used to determine correlations between the variables.

Patients were categorized as those with euthyroidism (normal TSH, FT3, and FT4), those with subclinical hyperthyroidism (decreased TSH along with normal FT3 and FT4), and those with hyperthyroidism (decreased TSH along with increased FT3 and FT4). Multivariable ordinal logistic regression models were used to assess the crude and adjusted (controlling for age, gender, and BMI) odds ratios (ORs) and 95% confidence intervals (95% CIs) for the relationships between hyperthyroidism (dependent variable) and trace elements. We also detected thyroid-specific antibodies (TPOAb and TgAb). According to TPOAb and/or TgAb, participants were also categorized as the negative antibody group (both TPOAb and TgAb were negative), the weakly positive antibody group (TPOAb and/or TgAb was positive, and both were <500 IU/ml) and the strongly positive antibody group (either TPOAb or TgAb was ≥500 IU/ml). Multivariable ordinal logistic regression models were used to assess the crude and adjusted ORs and 95% CIs for the relationships between thyroid-specific antibodies grade (dependent variable) and trace elements. In addition, bivariate analysis models were used to test the association between orbitopathy and trace elements. A *P* value < 0.05 was considered statistically significant, and all tests were two-tailed.

## 3. Results

### 3.1. Cohort Characterization and Thyroid Parameters

Of the 244 participants, 186 were female, and 58 were male. The characteristics of the different groups are shown in Table 1. No significant differences in age, gender, and BMI across the groups were found. Thyroid parameters (TSH, FT3, and FT4) in the EUGD group were similar to those in the NC group, but the thyroid-specific antibodies (TPOAb and TgAb) were higher than those in the NC group (both *P* < 0.001). However, compared with the NC and EUGD groups, the HyGD group showed a significant increase in FT3 and FT4 and significant decrease in TSH (all *P* < 0.001). The HyGD group also exhibited a higher level of TRAb compared with the EUGD group (*P* < 0.001). The GO group exhibited thyroid parameters similar to those in the NC group but TPOAb levels were higher than those in the NC group. In addition, TgAb levels in the GO group were significantly lower than those in the EUGD group. TRAb levels were not significantly different between the GO and EUGD groups.

Spearman correlation analysis of our patients revealed inverse relationships between TSH and TPOAb (*r* = −0.337, *P* < 0.001) and between TSH and TgAb (*r* = −0.320, *P* < 0.001). Positive correlations were also found between TRAb and FT3 (*r* = 0.349, *P* < 0.001) and between TRAb and FT4 (*r* = 0.227, *P* = 0.006).

### 3.2. Levels of Trace Elements in GD Patients

**Figure 1:**
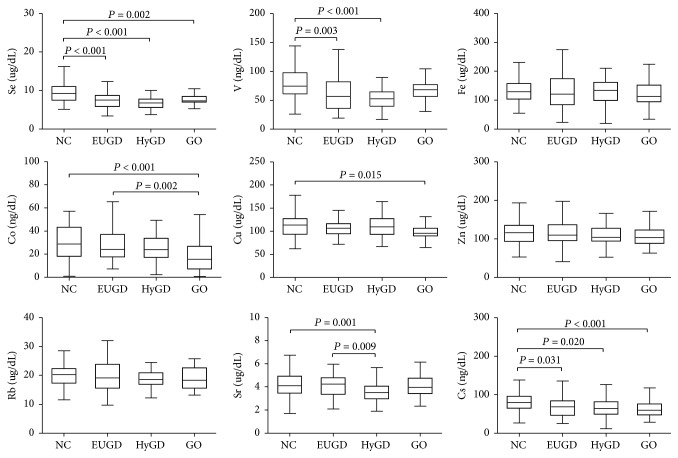
Comparison of trace elements levels. Data were expressed as median and interquartile intervals. Se, selenium; V, vanadium; Fe, iron; Co, cobalt; Cu, copper; Zn, zinc; Rb, rubidium; Sr, strontium; Cs, cesium; NC, normal controls; EUGD, subjects with stable euthyroid status or subclinical hyperthyroidism after treatment who used to be Graves' disease; HyGD, newly diagnosed Graves' disease patients; GO: Graves' ophthalmopathy patients with euthyroid state or subclinical hyperthyroidism after treatment with antithyroid drugs.

As shown in Figure 1, there were significant differences in serum trace elements. Serum Se (*P* < 0.001), V (*P* = 0.003), and Cs (*P* = 0.031) levels were significantly lower in EUGD group than in NC group, but serum Co, Cu, and Sr levels were not (Supplementary Table 1). Next, we compared the levels of serum trace elements in the HyGD and EUGD groups, which exhibit differences in thyroid function. We found that serum Sr was significantly lower in the HyGD group than in the EUGD group (*P* = 0.009). There were no difference in Se, V, Co, and Cs in the HyGD group compared with the EUGD group. The HyGD group had significantly lower levels of Se (*P* < 0.001), V (*P* < 0.001), Sr (*P* = 0.001), and Cs (*P* = 0.020) than the NC group.

### 3.3. Relationships between Trace Elements and Thyroid Parameters

To demonstrate the associations between serum trace elements concentrations of all the participants, we carried out Spearman correlation analysis on the whole population (Supplementary Table 2). Although the concentration of one trace element may be associated with other elements, no multicollinearity was detected according to the variance inflation factor statistics in multiple linear regression analysis models.

Table 2 shows Spearman correlation analysis between trace elements and thyroid parameters of all the participants. Serum Se was positively correlated with TSH (*r* = 0.254, *P* < 0.001) but negatively correlated with FT3 (*r* = −0.192, *P* = 0.006), FT4 (*r* = −0.173, *P* = 0.013), TPOAb (*r* = −0.161, *P* = 0.021), and TgAb (*r* = −0.237, *P* = 0.001). Serum V was negatively correlated with FT3 (*r* = −0.313, *P* < 0.001) and FT4 (*r* = −0.165, *P* = 0.018). Serum Cu was positively correlated with FT4 (*r* = 0.184, *P* = 0.008) but inversely correlated with TSH (*r* = −0.158, *P* = 0.024). Serum Sr was negatively correlated with FT3 (*r* = −0.180, *P* = 0.010) and FT4 (*r* = −0.244, *P* < 0.001) and positively correlated with TSH (*r* = 0.191, *P* = 0.006). Serum Cs was negatively correlated with TgAb (*r* = −0.155, *P* = 0.026).

Multivariable ordinal logistic regression models were used to assess the associations between hyperthyroidism or thyroid-specific antibodies grade with trace elements in the participants. Analyses unadjusted and adjusted for several confounders, including age, gender, and BMI, demonstrated that elevated serum Cu may be significantly associated with hyperthyroidism (OR = 1.025, 95% CI: 1.011–1.040, and *P* = 0.001 and OR = 1.031, 95% CI: 1.015–1.047, and *P* < 0.001, resp.), while lower serum Co and Rb were found to be associated with hyperthyroidism (OR = 0.988, 95% CI: 0.976–0.999, and *P* = 0.036 for Co and OR = 0.910, 95% CI: 0.839–0.988, and *P* = 0.024 for Rb) in the adjusted model (Table 3). Serum Se were negatively correlated with the levels of thyroid-specific antibodies (TPOAb and TgAb) in both the unadjusted and adjusted models (OR = 0.828, 95% CI: 0.734–0.933, and *P* = 0.002 and OR = 0.831, 95% CI: 0.736–0.939, and *P* = 0.003, resp.). However, serum Cu may be significantly associated with higher thyroid-specific antibodies grade (OR = 1.012, 95% CI: 1.001–1.023, and *P* = 0.032 and OR = 1.016, 95% CI: 1.004–1.028, and *P* = 0.007 for the unadjusted and adjusted models, resp.) (Table 4).

### 3.4. Trace Elements and Orbitopathy

To investigate the levels of trace elements in euthyroid patients with and without orbitopathy, we compared serum trace elements levels in the GO, EUGD, and NC groups. We found that serum Co levels were significantly lower in the GO group than in the EUGD group (*P* = 0.002), and Se (*P* = 0.002), Co (*P* < 0.001), Cu (*P* = 0.015), and Cs (*P* < 0.001) tended to be lower in the GO group than in the NC group. However, as shown in Figure 1, there were no significant differences in the levels of serum Fe, Zn, or Rb between the groups.

A bivariate analysis was performed to examine the association between orbitopathy and trace elements in the patients. We found that Co and Cu may negatively be associated with orbitopathy based on unadjusted model and the model adjusted for age, gender, and BMI (OR = 0.963, 95% CI: 0.940–0.987, and *P* = 0.003 and OR = 0.963, 95% CI: 0.938–0.988, and *P* = 0.004 for Co; OR = 0.977, 95% CI: 0.961–0.993, and *P* = 0.004 and OR = 0.976, 95% CI: 0.958–0.995, and *P* = 0.011 for Cu, resp.) (Table 5).

## 4. Discussion

To the best of our knowledge, this is the first study to investigate the serum trace elements in patients with GD living in the region with adequate iodine intake in Northeast China.

In the present study, we found that serum Se levels in patients with pathological thyroid conditions, including euthyroid GD patients, newly diagnosed GD patients with hyperthyroidism, and GO patients with euthyroidism, were significantly lower, to varying degrees, than those in normal controls. Serum Se levels in our healthy controls (9.20 *μ*g/dL, 92.0 *μ*g/L), who resided in areas with marginal soil Se content [21], were similar to the levels found throughout most of Europe, including Denmark (99.8 *μ*g/L) [22] and Greece (91.8 *μ*g/L) [23]. Kucharzewski et al. [24] demonstrated that patients with GD have lower serum Se levels, which is in line with our findings. In the present study, the Se concentrations in the EUGD and HyGD groups were lower than those in the NC group, but there was no difference in Se concentrations between the HyGD group and EUGD group. A retrospective study reported that serum Se levels in GD patients who achieved remission after treatment with antithyroid drugs were higher than those in patients with a relapse, and high serum Se levels (>120 *μ*g/L) were beneficial to the outcome of GD patients [25]. Another study showed that Se supplementation can improve biochemical restoration and that levels of Se were negatively correlated with FT3 in GD [26].

In the present study, we demonstrated that serum Se levels were significantly lower in GO patients than in normal controls, which is in agreement with a study in Germans [27]. Rotondo Dottore et al. [28] found that Se may weaken the effects of oxidative stress, decrease the secretion of proinflammatory cytokines, and reduce the release of hyaluronic acid in orbital fibroblasts from GO patients. A randomized, double-blind, placebo-controlled trial conducted by the European Group on Graves' Orbitopathy (EUGOGO) found that supplementation with Se in patients with mild Graves' orbitopathy may improve the quality of life and retard the development of the disease [29], although the serum Se concentrations were not measured in this study. These results support the hypothesis that GD and GO patients are at risk of low serum* Se* levels; but further research is needed to identify the exact effect and its mechanism of action.

A high correlation between low serum Se status and a high degree of thyroid autoimmunity has been reported in several publications [30, 31], and our result is consistent with these findings. An epidemiological study conducted by Wu et al. [31] in two regions of China that were defined as having adequate Se content and low Se content indicated that higher serum Se levels were significantly associated with lower risk of autoimmune thyroiditis. A recent systematic review conducted by Wichman et al. [32] reported that Se supplementation may reduce thyroid-specific antibodies levels. Consequently, low serum Se is a trigger or a risk factor for abnormal thyroid function.

One study in a general US population conducted by Jain, based on the data from the 2011 to 2012 US National Health and Nutrition Examination Survey (NHANES), demonstrated that serum Cu was positively associated with FT4 and TT4 in men [33]. Consistent with this, our results indicated that Cu levels were slightly higher in patients with GD than in patients with EUGD (*P* = 0.261) and were positively associated with FT4 levels. In addition, we also found hyperthyroidism may be associated with higher Cu levels. There are several publications reporting that Cu metabolism was affected by hyperthyroidism [34, 35]. A study in animals conducted by Mittag et al. [36] found significant increase of Cu levels when treated with thyroid hormone. The decline expression of competing intracellular Cu-binding proteins and enhancive synthesis and export of hepatic ceruloplasmin, the major Cu-carrying protein, could explain this phenomenon.

There are conflicting reports on the relationship between serum Cu and thyroid autoimmunity. Erdal et al. [37] found no difference between Hashimoto's thyroiditis (HT) and normal controls. But a recent study conducted by Rasic-Milutinovic et al. [38] detected the higher blood Cu concentration in HT patients compared with normal controls. In the present study, we found higher Cu levels may be related with thyroid autoimmunity according to multivariable ordinal logistic regression. It was reported that the destruction of serum Cu homeostasis may contribute to the oxidative stress state in HT patients [38]. In addition, it has been detected that the activation of Fas/FasL (Fas ligand) could contribute to the pathogenesis of AITD. The accumulation of Cu in the liver may also promote the translation of FasL in patients with Wilson's disease and activate the system of Fas/FasL to subsequently induce apoptosis in liver cells [39]. It is reasonable to propose the same pathogenesis in thyroid autoimmunity, but further studies are needed to clarify.

Unlike serum Se, serum Cu in patients with GO is rarely studied. Here, we found that GO patients are at risk of a low serum Cu levels. Cu is integrated in the active center of superoxide dismutase (SOD), the essential antioxidant enzyme for the removal of the oxygen free radical and the prevention of oxidative stress damage. A study in an animal model suggested that the generation and accumulation of reactive oxygen species in the thyroid may disrupt the antioxidation system [40], and oxygen free radicals may simultaneously cause the proliferation of orbital fibrosis [41]. Kaur et al. [42] reported significantly reduced SOD levels in the plasma of hyperthyroid patients with GO, and there was no improvement even after treatment with antithyroid drugs. However, SOD activity was not assessed in the present study; therefore, further research is needed to determine the exact mechanism.

In the present study, we also detected the serum levels of several elements, including V, Fe, Co, Zn, Sr, and Cs. Although little is known about the biological function of these elements in thyroid diseases, the present study demonstrated the unique elements profile of those patients.

Our study had some limitations. One major limitation was its cross-sectional design. As a result of this design, we failed to observe any changes in trace elements in the same patient with different thyroid function and thyroid autoimmunity. Another limitation is that we did not measure serum and urinary iodine levels, although all of the participants resided in an area with adequate iodine levels [43].

In conclusion, trace elements, which are involved in various physiological and pathological processes, are closely related to the thyroid gland. The present study demonstrated lower serum Se levels in patients with GD, with or without ophthalmopathy. A relatively high level of serum Cu may be associated with hyperthyroidism. Our findings are also the first to indicate that a relatively low level of serum Cu may be associated with ophthalmopathy. However, further research must be conducted to elucidate the regulation of Cu homeostasis in thyroid diseases.

## Figures and Tables

**Table 1:** Demographics characteristics and thyroid parameters in different groups.

Variables	NC (*n* = 66)	EUGD (*n* = 55)	HyGD (*n* = 66)	GO (*n* = 57)
Age (years)^a^	42.3 ± 12.6	37.1 ± 12.6	38.6 ± 13.1	38.5 ± 12.1
Gender (F/M)	48/18	48/7	49/17	41/16
BMI (kg/m^2^)^a^	24.08 ± 2.84	23.31 ± 1.56	23.42 ± 2.18	22.99 ± 2.76
TSH (mIU/L)^b^	1.84 (1.16–2.90)	1.45 (0.24–2.41)	0.01 (0.00–0.02)^*∗*, #^	2.19 (0.82–3.66)
FT3 (pmol/L)^b^	4.46 (4.14–4.76)	4.81 (4.29–5.24)	14.39 (7.55–30.52)^*∗*, #^	4.35 (3.74–4.83)
FT4 (pmol/L)^b^	12.88 (11.97–13.81)	12.44 (11.15–14.12)	21.89 (15.84–35.98)^*∗*, #^	12.68 (11.11–14.12)
TPOAb (IU/mL)^b^	0.38 (0.13–1.07)	56.72 (8.87–223.00)^*∗*^	109.75 (18.21–447.38)^*∗*^	19.46 (0.13–203.48)^†^
TgAb (IU/mL)^b^	1.24 (0.92–3.85)	82.00 (27.09–209.70)^*∗*^	97.01 (18.49–367.50)^*∗*^	4.23 (1.22–33.96)^#^
TRAb (IU/mL)^b^	-	2.05 (1.24–6.72)	12.25 (5.30–21.02)^#^	5.98 (1.65–15.36)

EUGD: subjects with stable euthyroid status or subclinical hyperthyroidism after treatment who used to have Graves' disease; HyGD: newly diagnosed Graves' disease patients; GO: Graves' ophthalmopathy patients with euthyroid state or subclinical hyperthyroidism after treatment with antithyroid drugs; NC: normal controls. ^a^Results are expressed as mean ± SD. ^b^Results are expressed as medians (interquartile range); ^*∗*^*P* < 0.001 versus NC, ^†^*P* < 0.01 versus NC, and ^#^*P* < 0.001 versus EUGD; “-”: did not detect; TSH, thyroid stimulating hormone; FT3, free T3; FT4, free T4; TPOAb, thyroperoxidase antibody; TgAb, antithyroglobulin antibody; TRAb, TSH receptor autoantibody.

**Table 2:** Spearman correlation analysis between trace elements levels and thyroid parameters in all the participants, shown as correlation coefficient (*P* value).

Variables	TSH (*P*)	FT3 (*P*)	FT4 (*P*)	TPOAb (*P*)	TgAb (*P*)
Se	0.254 (0.000)	−0.192 (0.006)	−0.173 (0.013)	−0.161 (0.021)	−0.237 (0.001)
V	0.106 (0.129)	−0.313 (0.000)	−0.165 (0.018)	−0.127 (0.069)	−0.112 (0.109)
Fe	−0.034 (0.632)	0.128 (0.066)	−0.010 (0.884)	0.036 (0.609)	−0.025 (0.725)
Co	0.055 (0.431)	−0.034 (0.631)	−0.048 (0.492)	−0.038 (0.585)	−0.007 (0.926)
Cu	−0.158 (0.024)	0.122 (0.081)	0.184 (0.008)	0.038 (0.587)	0.082 (0.241)
Zn	0.022 (0.758)	0.093 (0.184)	−0.037 (0.597)	0.098 (0.164)	0.129 (0.065)
Rb	0.013 (0.854)	0.009 (0.897)	−0.053 (0.447)	−0.062 (0.376)	−0.006 (0.928)
Sr	0.191 (0.006)	−0.180 (0.010)	−0.244 (0.000)	−0.074 (0.290)	−0.124 (0.076)
Cs	0.110 (0.116)	−0.021 (0.767)	−0.061 (0.386)	−0.077 (0.274)	−0.155 (0.026)

Se, selenium; V, vanadium; Fe, iron; Co, cobalt; Cu, copper; Zn, zinc; Rb, rubidium; Sr, strontium; Cs, cesium; TSH, thyroid stimulating hormone; FT3, free T3; FT4, free T4; TPOAb, thyroperoxidase antibody; TgAb, antithyroglobulin antibody.

**Table 3:** Multivariable ordinal logistic regression to explore the association between hyperthyroidism and trace elements.

	Unadjusted *β* (95% CI)	Unadjusted OR (95% CI)	*P *value	Adjusted *β* (95% CI)^a^	Adjusted OR (95% CI)^a^	*P* value
Se	−0.059 (−0.206,0.087)	0.942 (0.814,1.091)	0.427	−0.052 (−0.200,0.095)	0.949 (0.819,1.100)	0.487
V	0.003 (−0.005,0.012)	1.003 (0.995,1.011)	0.455	0.005 (−0.004,0.014)	1.005 (0.996,1.014)	0.313
Fe	0.005 (0.000,0.011)	1.005 (1.000,1.011)	0.043	0.004 (−0.002,0.009)	1.040 (0.998,1.009)	0.158
Co	−0.012 (−0.023,0.000)	0.988 (0.977,0.999)	0.042	−0.012 (−0.024, −0.001)	0.988 (0.976,0.999)	0.036
Cu	0.025 (0.011,0.039)	1.025 (1.011,1.040)	0.001	0.030 (0.015,0.046)	1.031 (1.015,1.047)	0.000
Zn	−0.004 (−0.015,0.007)	0.996 (0.985,1.007)	0.454	−0.002 (−0.014,0.009)	0.998 (0.987,1.009)	0.705
Rb	−0.075 (−0.149, −0.002)	0.927 (0.862,0.998)	0.045	−0.094 (−0.175, −0.012)	0.910 (0.839,0.988)	0.024
Sr	−0.173 (−0.443,0.096)	0.841 (0.642,1.101)	0.207	−0.287 (−0.580,0.005)	0.750 (0.560,1.005)	0.054
Cs	0.010 (−0.003,0.022)	1.010 (0.997,1.022)	0.137	0.012 (−0.001,0.025)	1.012 (0.999,1.025)	0.064

^a^Adjusted for age, gender, and BMI; Se, selenium; V, vanadium; Fe, iron; Co, cobalt; Cu, copper; Zn, zinc; Rb, rubidium; Sr, strontium; Cs, cesium; OR: odds ratio; 95% CI: 95% confidence interval.

**Table 4:** Multivariable ordinal logistic regression to explore the association between thyroid-specific antibody grade and trace elements.

	Unadjusted *β* (95% CI)	Unadjusted OR (95% CI)	*P* value	Adjusted *β* (95% CI)^a^	Adjusted OR (95% CI)^a^	*P* value
Se	−0.189 (−0.310, −0.069)	0.828 (0.734, 0.933)	0.002	−0.185 (−0.307, −0.063)	0.831 (0.736, 0.939)	0.003
V	−0.001 (−0.008, 0.006)	0.999 (0.992, 1.006)	0.857	0.000 (−0.007, 0.007)	0.999 (0.993, 1.007)	0.978
Fe	0.003 (−0.001, 0.007)	1.003 (0.999, 1.007)	0.146	0.002 (−0.003, 0.006)	1.002 (0.997, 1.006)	0.447
Co	0.001 (−0.006, 0.008)	1.006 (0.994, 1.008)	0.858	0.000 (−0.007, 0.008)	1.003 (0.993, 1.008)	0.928
Cu	0.012 (0.001, 0.023)	1.012 (1.001, 1.023)	0.032	0.016 (0.004, 0.028)	1.016 (1.004, 1.028)	0.007
Zn	0.002 (−0.006, 0.010)	1.002 (0.994, 1.010)	0.589	0.001 (−0.007, 0.009)	1.002 (0.993, 1.009)	0.801
Rb	−0.019 (−0.070, 0.031)	0.981 (0.933, 1.032)	0.453	−0.025 (−0.077, 0.026)	0.975 (0.926, 1.026)	0.331
Sr	−0.054 (−0.262, 0.154)	0.947 (0.769, 1.066)	0.609	−0.062 (−0.278, 0.154)	0.940 (0.757, 1.166)	0.573
Cs	0.000 (−0.010, 0.009)	1.000 (0.991, 1.009)	0.959	0.001 (−0.009, 0.010)	1.001 (0.991, 1.010)	0.910

^a^Adjusted for age, gender, and BMI; Se, selenium; V, vanadium; Fe, iron; Co, cobalt; Cu, copper; Zn, zinc; Rb, rubidium; Sr, strontium; Cs, cesium; OR: odds ratio; 95% CI: 95% confidence interval.

**Table 5:** Bivariate analysis to examine the association between Graves' ophthalmopathy and trace elements levels.

	Unadjusted OR (95% CI)	*P* value	Adjusted OR (95% CI)^a^	*P* value
Se	1.058 (0.921–1.214)	0.426	1.117 (0.940–1.328)	0.210
V	1.000 (0.992–1.008)	0.463	1.008 (0.997–1.019)	0.152
Fe	0.996 (0.990–1.001)	0.110	0.992 (0.985–1.000)	0.051
Co	0.963 (0.940–0.987)	0.003	0.963 (0.938–0.988)	0.004
Cu	0.977 (0.961–0.993)	0.004	0.976 (0.958–0.995)	0.011
Zn	0.993 (0.983–1.003)	0.186	1.000 (0.988–1.012)	0.956
Rb	0.996 (0.947–1.047)	0.874	1.004 (0.941–1.070)	0.910
Sr	1.061 (0.831–1.355)	0.633	1.167 (0.876–1.553)	0.291
Cs	0.991 (0.979–1.003)	0.147	0.994 (0.978–1.010)	0.450

^a^Adjusted for age, gender, and BMI; Se, selenium; V, vanadium; Fe, iron; Co, cobalt; Cu, copper; Zn, zinc; Rb, rubidium; Sr, strontium; Cs, cesium; OR: odds ratio; 95% CI: 95% confidence interval.

## Supplementary Materials

Supplementary Materials Supplementary Table 1: serum trace elements concentrations in different groups. Supplementary Table 2: Spearman correlation analysis among serum concentrations of the different trace elements in all the participants.
