# Supplementary material for: Serum Trace Elements Profile in Graves' Disease Patients with or without Orbitopathy in Northeast China
Source: Biomed Res Int. 2018 Jan 10;2018:3029379. doi: 10.1155/2018/3029379 (PMC5818896; doi:10.1155/2018/3029379)
Supplement: Supplementary Materials — Supplementary Table 1: serum trace elements concentrations in different groups. Supplementary Table 2: Spearman correlation analysis among serum concentrations of the different trace elements in all the participants. [file 3029379.f1.doc]

**Supplementary Material**

Supplementary Table 1. Serum trace elements concentrations in different groups.

| Variables | NC (n=66)  Median (P25-P75) | EUGD (n=55)  Median (P25-P75) | HyGD (n=66)  Median (P25-P75) | GO (n=57)  Median (P25-P75) |
| --- | --- | --- | --- | --- |
| Se (µg /dl) | 9.20 (7.47-11.03) | 7.53 (5.84-8.76) | 6.76 (5.56-7.78) | 7.40 (6.96-8.44) |
| V (ng /dl) | 74.54 (60.85-97.68) | 56.69 (36.30-81.96) | 52.65 (39.95-64.63) | 68.14 (56.87-76.92) |
| Fe (µg /dl) | 129.09 (103.34-157.71) | 120.71 (83.82-174.91) | 134.38 (98.17-162.07) | 113.17 (94.24-151.85) |
| Co (ng/dl) | 28.72 (18.06-43.33) | 24.09 (17.54-37.03) | 23.97 (17.07-33.68) | 15.59 (7.09-26.84) |
| Cu (µg /dl) | 113.59 (93.18-127.44) | 106.53 (94.30-116.49) | 109.39 (92.92-127.18) | 95.93 (89.92-106.73) |
| Zn (µg /dl) | 116.49 (92.77-135.44) | 109.97 (95.55-136.83) | 104.32 (93.96-127.85) | 103.53 (88.02-123.30) |
| Rb (µg /dl) | 20.24 (17.26-22.32) | 19.12 (15.51-23.78) | 18.62 (16.84-20.90) | 18.31 (15.51-22.62) |
| Sr (µg /dl) | 4.10 (3.45-4.93) | 4.24 (3.34-4.78) | 3.49 (2.96-4.06) | 3.96 (3.41-4.75) |
| Cs (ng/dl) | 79.87 (65.15-96.14) | 68.89 (46.25-84.09) | 64.62 (49.27-82.23) | 60.08 (47.59-75.92) |

Data are shown as medians (interquartile range). Se, selenium; V, vanadium; Fe, iron; Co, cobalt; Cu, copper; Zn, zinc; Rb, rubidium; Sr, strontium; Cs, caesium; NC: normal controls; EUGD: subjects with stable euthyroid status or subclinical hyperthyroidism after treatment who used to be Graves’ disease; HyGD: new diagnosed Graves’ disease patients; GO: Graves’ ophthalmopathy patients with euthyroid state or subclinical hyperthyroidism after treatment with antithyroid drugs.

Supplementary Table 2. Spearman correlation analysis among serum concentrations of the different trace elements in all the participants.

| Variables | Se(*P*) | V(*P*) | Fe(*P*) | Co(*P*) | Cu(*P*) | Zn(*P*) | Rb(*P*) | Sr(*P*) |
| --- | --- | --- | --- | --- | --- | --- | --- | --- |
| V | 0.221 (0.001) |  |  |  |  |  |  |  |
| Fe | 0.238 (0.000) | 0.149 (0.020) |  |  |  |  |  |  |
| Co | 0.120 (0.062) | 0.177 (0.006) | 0.140 (0.029) |  |  |  |  |  |
| Cu | 0.170 (0.008) | 0.033 (0.614) | -0.036 (0.582) | 0.204 (0.001) |  |  |  |  |
| Zn | 0.149 (0.020) | 0.028 (0.666) | 0.235 (0.000) | 0.216 (0.001) | 0.285 (0.000) |  |  |  |
| Rb | 0.352 (0.000) | 0.184 (0.004) | 0.363 (0.000) | 0.071 (0.273) | 0.228 (0.000) | 0.209 (0.001) |  |  |
| Sr | 0.271 (0.000) | 0.333 (0.000) | 0.139 (0.031) | 0.228 (0.000) | 0.060 (0.350) | 0.216 (0.001) | 0.116 (0.071) |  |
| Cs | 0.377 (0.000) | 0.201 (0.002) | 0.180 (0.005) | 0.139 (0.030) | 0.217 (0.001) | 0.144 (0.025) | 0.581 (0.000) | 0.146 (0.023) |

Se, selenium; V, vanadium; Fe, iron; Co, cobalt; Cu, copper; Zn, zinc; Rb, rubidium; Sr, strontium; Cs, cesium. Data were shown as correlation coefficient (*P* value).
